# Supplementary material for: Are carbon emissions trading and green financial instruments synergistic? -Comprehensive quantitative research based on content analysis
Source: PLoS One. 2024 Mar 7;19(3):e0298601. doi: 10.1371/journal.pone.0298601 (PMC10919601; doi:10.1371/journal.pone.0298601)
Supplement: S1 Data — (ZIP) [file pone.0298601.s001.zip › Data/地级市Z_F工作报告.zip!/╣π╢1⁄2/╢1⁄2▌╕╩╨/└┤╫╘╣1⁄2╓┌║┼í╛┬φ┐╦╩2╛▌═°í┐_2019.docx]

各位代表：

现在，我代表东莞市人民政府向大会作政府工作报告，请予审议，并请各位政协委员和其他列席人员提出意见。

2018年工作回顾

过去一年，面对复杂经济形势带来的严峻挑战，在省委省政府和市委的正确领导下，市政府坚持以习近平新时代中国特色社会主义思想为指导，全面贯彻党的十九大精神，深入学习贯彻习近平总书记对广东重要讲话精神，认真落实省委“1+1+9”等各项决策部署，强化执行、克难奋进，统筹推进稳增长、促改革、调结构、惠民生、防风险等各项工作，经济社会发展保持稳中有进、进中向好的良好态势。全市生产总值突破8000亿元大关，预计达到8300亿元，同比增长7.5%左右，快于全国全省。进出口总额突破1.3万亿元，排名全国第5。规上工业企业突破1万家，排名全省第1。市场主体突破115万户，排名全省地级市第1。各项税收总额突破2300亿元，排名全省第3。实现市一般公共预算收入650亿元，排名全省第4。各项存款余额突破1.4万亿元，增速全省第1。虎门港综合保税区获批，东莞成为国家跨境电子商务综合试验区。28个镇全部入围全国综合实力千强镇前400强，百强镇增至15个。村组两级纯收入突破150亿元，增长14.5%。东莞被评为“全国社会治理创新示范市”，首获中国法治政府奖。在人民大学、社科院等权威机构发布的相关报告中，东莞的政商关系健康指数、“互联网+”指数、综合经济竞争力分别排名全国第1位、第8位和第13位，民生发展指数排名全国地级市第3位。东莞发展的活力、品质和魅力不断提升。具体来说，我们抓好了以下工作：

一年来，我们坚持提高政治站位，坚决贯彻落实习近平新时代中国特色社会主义思想

在市委的统一部署下，把学习贯彻落实习近平新时代中国特色社会主义思想作为全市政府系统的首要政治任务，努力做到学懂、弄通、做实。我们认真学习贯彻总书记在全国“两会”参加广东代表团审议重要讲话精神，将其与“三个定位、两个率先”和“四个坚持、三个支撑、两个走在前列”等重要指示批示精神一体学习领会、整体贯彻落实，努力争当全省实现“四个走在全国前列”排头兵。我们认真学习贯彻总书记视察广东重要讲话精神，把参与粤港澳大湾区建设作为东莞深化改革开放的“纲”，研究制定实施方案和三年行动计划，依托滨海湾新区、中子科学城等，加快打造对接湾区的重大平台，努力在湾区合作发展中抢占先机。我们认真学习贯彻习近平新时代中国特色社会主义经济思想，主动适应高质量发展的要求，科学做好产业发展谋划，掀起产业招商新热潮，切实加大对非公经济的扶持力度，努力推动以先进制造业为核心的实体经济稳步发展。我们认真学习贯彻习近平生态文明思想，坚决打好治水攻坚战、蓝天保卫战、净土防御战和固废处理处置攻坚战，加快解决突出环境问题，认真补齐短板，改善生态环境，提升发展质量。我们认真学习贯彻总书记关于实施乡村振兴战略的重要讲话精神，按照产业兴旺、生态宜居、乡风文明、治理有效、生活富裕的总要求，明确东莞乡村振兴的时间表和路线图，努力推动农业全面升级、农村全面进步、农民全面发展。我们认真学习贯彻总书记关于做好督察工作的重要指示精神，全力做好中央和省在安全生产、扫黑除恶、海洋等方面督察巡视的迎检和整改工作，做到真查摆、真落实、真整改，得到了督察组的充分肯定。全市政府系统“四个意识”切实增强，思想自觉和行动自觉进一步强化。习近平新时代中国特色社会主义思想的光辉照耀着莞邑大地，指引着前进方向。

一年来，我们坚持发展第一要务，全力推动东莞经济高质量发展

大力抓好产业谋划招引和项目落地建设。着眼于推动产业长远健康发展，出台重点新兴产业发展规划，确定五大新兴领域和十大重点产业，规划“一核三带十区”，布局经济发展新增长点。组建市级新型招商机构，建立市镇联合、资源统筹、跟踪洽谈等新机制，出台“重特大项目招商十条”、“高质量利用外资十条”、促进总部经济发展、扶持企业上市等新政策。召开全市规模最大、规格最高的招商大会，集中签约重特大项目29个，涉及金额超过4000亿元。13名市领导挂点服务22个五亿元以上项目，推动加快落地。全市新引进亿元以上内资项目172宗，协议金额2052亿元；引进千万美元以上外资项目102宗，协议金额36.5亿美元。成立建设工程总指挥部，开展“百日攻坚”大会战，完成重大项目投资631.4亿元，增长22.9%。

大力推动企业倍增发展。出台“高质量倍增十条”，调整确定市级试点企业286家，选定协同倍增企业910家，创新推出全流程诊断、“倍增卡”等举措，解决问题700多项，新增用地指标570多亩，倍增企业主营业务收入增长16%以上。着力打造智能制造全生态链，建立资源服务池，建成示范线109条，资助改造项目311个。推动中小微企业做大做强，全市新增“四上”企业3800家、“小升规”企业2784家，增量均为全省第1。完成规上工业增加值3900亿元，增长6.5%左右。其中，先进制造业、民营工业占比升至52.3%和49.6%。单位GDP能耗下降6%左右。

大力优化非公经济发展环境。强化对企服务意识，市镇领导班子挂点服务企业，设立首席服务官，实施政策告知推送机制，主动向企业推送政策、服务和举措。召开非公经济代表人士座谈会，认真听取企业呼声，邀请700多名企业家参加全市非公经济大会，围绕减轻企业负担、促进企业转型、拓展企业发展空间等方面，出台50条160项扶持政策。成功举办世界莞商大会，1200多名海内外莞商汇聚一堂，共谋发展。成立上市莞企发展投资基金，为上市企业协调化解各类债务风险20亿元。全年为企业减免税收248亿元以上。

一年来，我们坚持创新第一动力，加快建设国家创新型城市

加快推进区域创新。全面启动广深港澳科技创新走廊（东莞段）建设，加快实施133个沿线环境品质提升项目，出台实施空间规划，筛选出260个总投资3600多亿元的入库科技项目，努力在对接广深港澳创新资源上占得先机。散裂中子源顺利通过国家验收，填补了国内脉冲中子应用领域的空白。全面启动中子科学城建设，省政府同意将其上升为省级发展平台，并以中子科学城和深圳光明科学城、港深落马洲河套地区为核心载体创建综合性国家科学中心。与中科院高能所共同谋划建设南方光源，松山湖材料实验室和粤港澳交叉科学中心建设取得扎实进展。成功举办粤港澳大湾区院士峰会暨第四届广东院士高峰年会，为发展院士经济打下坚实基础。

加快聚集创新要素。狠抓“技能人才之都”建设，实施劳动力技能素质提升三年行动计划，着力建设知识型、技能型、创新型劳动者大军。实施高企“树标提质”行动计划，高企总量预计达5798家，居全省地级市第1。新增博士后、博士工作平台30家。入选省创新科研团队6个，获省财政资助金额达1.8亿元，创历史之最。专利申请量和授权量预计分别增长20%和40%。PCT国际专利申请量跃居全省第2。获中国专利奖银奖4项。R&D投入占比预计达2.55%。

加快推动科技、金融、产业“三融合”。强化财政和国有资本对创新的引领，设立规模100亿元以上的产业投资基金和并购基金，整合成立科技创新金融集团，贴息发放“三融合”贷款68亿元。强化金融对实体经济的支撑，制造业、小微企业贷款分别突破1000亿元、2000亿元，“倍增计划”和高企贷款均突破300亿元。专利权质押融资金额规模全省第2。强化对资本市场发展的扶持。新增境内外上市企业2家、上市后备企业33家。东莞成为全省唯一齐备港澳台资银行的地级市。

一年来，我们坚持深化改革开放，努力争创东莞改革发展新优势

着力抓好重点领域改革。把改革作为推动发展的关键一招，坚持问题导向、结果导向，谋划推进了一系列重大改革，有效破解了一大批制约发展的历史遗留问题。深化不动产登记改革，抵押和转移登记从原来的数月分别缩短到3个和5个工作日办结；深化项目审批制度改革，推行告知承诺信任审批模式，审批项目从162项压缩到60项，从8月份起审批时间减少一半以上；深化代建制改革，推动深度市场化、专业化代建，提升财政投资项目建设水平和速度；深化新型产业用地改革，出台工业大厦分割销售、5年税收奖励等工改M0特惠政策，促进新型产业发展；深化招投标改革，改进施工招标项目中标方式，提高招投标效率和择优力度；深化商事制度改革，以构建“一平台、三工程”市场监管体系为牵引，创新事中事后监管方式，实现开办企业便利度连续三年全省地级市第1；深化市属国企改革，整合组建新的交投和水务集团，更好地发挥其引领发展和服务城市的功能；创新统筹开发模式和利益平衡机制，妥善解决东部工业园（企石辖区）历史遗留问题，6520亩土地纳入松山湖统筹。

着力提升对外开放水平。出台促进外经贸稳定发展20条举措，积极应对国际贸易形势变化，全年进出口增长10%以上，较好地帮助企业在严峻形势下稳定了生产经营。东莞连续两年位列“外贸百强城市”第3位。大力培育新模式、新业态，跨境电商进出口350亿元，增长120%，总量全国第1。保税物流进出口2300亿元，增长15%。广东（石龙）铁路国际物流基地启用多式联运业务。东莞始发中欧班列货运量增长42%。大力开拓国内外市场，搭建“莞货全国行”平台，开展“乐购东莞”等促消费活动，成功举办海丝博览会、加博会、漫博会、智博会、台博会，社会消费品零售总额增长8.1%左右。

着力推动重大平台建设。松山湖实现生产总值630亿元，增长13.9%；完成固投182.2亿元，增长22.6%；协议引资150亿元，增长29.3%，对片区带动作用进一步增强。滨海湾新区建设纳入大湾区国家战略，发展总体规划、城市总体规划通过审议，“二横五纵”骨干路网加快推进，两个滩涂开发项目实质性动工，沙角电厂两台燃煤机组关停，紫光芯云产业城、OPPO智能制造中心等项目加快推进。水乡新城片区生产总值增速快于全市0.3个百分点。水乡新城控规完成公示，启动区项目加快推进。银瓶新区完成海绵城市规划编制，粤海产业园一期建成开园。

着力提升合作发展水平。加强对口帮扶。援疆援藏工作扎实推进，兵团草湖产业园纺织项目高效建设运营，支持当地设立规模8亿元的产业发展基金；打造出林芝布久乡小康示范镇等精品项目，7个镇街与巴宜区7个乡镇结对；新增16个镇街对口帮扶云南昭通6个县区，捐赠清洁能源公交车200辆；与黑龙江牡丹江市缔结友好城市，推动20.7亿元的21个产业合作协议落地；顺利完成对口帮扶韶关十大行动计划，多项工作排名全省前列；落实韶关、揭阳精准扶贫精准脱贫资金4.68亿元，推动323个贫困村集体经济收入增长51%，预脱贫14054户、40278人。加强对内合作。加快推进深莞惠经济圈（3+2）和穗莞深化合作系列事项，并就轨道互通、环保共治等与广州、深圳进行了深入协商。举办近年来规模最大的莞港产业合作推介会，签订项目39个，金额350亿元。全方位加强对外交往。汤加国王到莞访问，美国利百伦市、韩国牙山市、德国乌波塔尔市等友城代表团来莞交流，友好关系持续深化。

一年来，我们坚持建设美丽东莞，加快提升城市品质和内涵

大力推进城市基础设施建设。实施城市品质三年提升计划，全面启动总投资1689.9亿元的582个重点项目，完工200个。东莞城市总体规划（2016-2035年）报国务院审批。大力推进轨道交通建设，积极争取将广深第二高铁纳入大湾区近期建设项目，2号线三期、3号线一期工程前期工作进展顺利。协调广铁集团在虎门站新增20多趟列车停靠，市民无需辗转便可直接乘高铁至北京、上海。大力推进高快速路建设，环莞快速二期、虎门二桥主线桥全线贯通，环莞三期华为段动工。莞番高速、深圳外环高速东莞段等加快建设。更新改造供水管网433.2公里，新建天然气管道约300公里，完成电网投资30亿元。

大力推进环境污染治理。成立污染防治攻坚战指挥部，出台三年行动计划。狠抓水污染治理。新建截污管网1525公里，通水1427公里。完成102条污染河涌整治，推进6家污水厂新建扩建、35家提标改造。开展河湖管护三大专项行动，全面铺开入河、入海排污口整治。清理整治砂场182个。茅洲河、石马河流域水质好转，综合污染指数分别下降55.5%和19.7%。7个国省考断面水质总体改善。狠抓大气污染治理。淘汰整治“散乱污”企业13073家，治理VOCs企业4870家，压减煤炭消费量118.8万吨。更换纯电动公交车2920辆。PM2.5年均浓度下降2.7个百分点。狠抓固废处理处置。开展“利剑一号”行动，严厉打击固废偷运、非法跨市转移等行为。完成10座垃圾填埋场综合整治，建成东南部卫生填埋场（一期），建筑垃圾资源化实现零突破。狠抓土壤污染防治，完成三个修复治理试点示范工程。

大力推进“两违”治理。坚持露头就打、出土就拆，对新增违建、“小产权房”实行“零容忍”、“零增量”。全市拆除（整改）新增违建98.9万平方米，治理违建1060万平方米，超额完成省下达任务。建立历史遗留违法建筑信息数据库，完善4868亩用地手续。盘活存量土地10365亩，处置闲置土地4251亩，复耕复绿土地3500多亩，有效拓展了发展空间。

大力推进城市更新改造。出台关于深化改革全力推进城市更新提升城市品质的意见，梳理出总面积1.9万亩的30个更新单元向外推介，积极打造长安科技商务区、东城牛山钟屋围、厚街楷模家居等亮点。全年投入208亿元，完成“三旧”改造6880亩，新增实施改造9837亩，力度历年最大，受省奖励用地指标3844亩。加快推进轨道交通TOD综合开发，调整优化在库储备土地规划。松山湖北站推出首期出让地块，虎门高铁站用地补偿基本完成，45个站场TOD综合开发规划编制抓紧推进。

大力提升城市精细化管理水平。全市上下众志成城，各级领导干部靠前指挥，成功抵御40多年来影响最严重的台风“山竹”，最大程度降低了灾害损失，迅速恢复了生产生活秩序。高度重视群众反映强烈的交通拥堵问题，设立综合交通运输联席会议，完成治理第一批交通拥堵节点12个，新建人行天桥13座，全市交通拥堵状况有所改善，虎门大桥疏堵保畅成效明显。开展城市精细化管理考核，查处城市“六乱”28万宗。整治易涝点15个。黄江、樟木头、麻涌、洪梅、望牛墩成功创建省森林小镇。东莞植物园一期建成开园，市民亲近自然、感受绿色更加便捷。

大力实施乡村振兴战略。出台“1+1+N”政策体系，围绕农村环卫、基础设施建设等重点，高标准推进63个亮点项目建设，全部村（社区）基本完成“三清理”、“三拆除”等环境整治任务。农村集体产权制度改革试点任务顺利完成。农村土地承包经营权确权登记颁证率达94%。次发达镇生产总值平均增速快于全市1个百分点。70个次发达村（社区）经营性纯收入增长14.7%。

一年来，我们坚持以人民为中心，致力满足市民群众日益增长的美好生活需要

加快推进教育扩容提质。编制实施新一轮学校建设规划。新增中小学幼儿园53所、学位近7万个，组建教育集团7个，规划建设未来学校3所。新招收随迁子女入读公办学位和补贴学位15.6万人，增加129.1%。向民办学校发放教师从教津贴、普惠性幼儿园补助等1.8亿元。给予困难学生助学减免1.16亿元。高考本科上线率达65.4%，创历史新高。12所中职学校实行中高职贯通培养。东莞理工学院获得硕士学位授予资格，实现零突破。

切实加大优质医疗资源供给。选定5所医院建设区域中心医院，常平医院、塘厦医院、麻涌医院纳入市属管理。完成200所社区卫生服务机构标准化建设，诊疗量占比升至28%。实施家庭医生签约服务，签约258万人。深入推进“医药分开”改革，实施药品零加成政策，为群众减负6350万元。开展健康讲座2300多场，“说医不二”微视频点击量突破1000万次。东莞公立医院综合改革成效明显，获得国务院办公厅督查激励。

着力加强公共安全防控体系建设。深入开展“扫黑除恶”专项斗争，打掉涉黑社会犯罪组织6个、恶势力犯罪团伙440个。社会治安实现“三降三升”，“两抢”立案数下降近八成、“飞抢”警情下降九成。组建公安铁骑，在交通疏堵、快速处警、震慑犯罪等方面成效明显。开展整治制毒物品非法流失问题专项行动，获公安部高度肯定。完成“二标四实”基础信息采集，基本摸清了全市实有人口、房屋、单位及设施等情况，为长治久安和精细化管理打下了坚实基础。大力推动安全生产“四个全覆盖”，深入开展建筑施工安全、有限空间作业、道路交通安全等专项治理行动，生产安全事故宗数下降14.8%，未发生重大及以上安全事故。深入实施消防安全“个十百千万”工程，顺利完成消防部队改制转隶。启动国家食品安全示范城市创建。做好非洲猪瘟防控。“智网工程”进入常态化运作、制度化管理，发现、处置各类隐患120.2万个。集中开展信访矛盾化解攻坚，有效维护了社会和谐稳定。

推动文化体育社保等各项事业繁荣发展。基层综合性文化服务中心实现全覆盖。举办“时代交响”音乐会、“时代印记”图片展，组织开展“看东莞”系列活动，营造庆祝改革开放40周年良好氛围。亚运会上获5项金牌，省运会东莞金牌总数居地级市第1，首届市民运动会成功举办。出台新的社会医疗保险办法，实行工伤和失业保险浮动费率管理。71家定点医院实现跨省异地联网结算。为特困、低保、困难群众发放各类救助1.5亿元。推进住房保障体系建设，解决6998户困难群众住房需求。做好房地产调控，推动平稳健康发展。“四经普”单位清查圆满收官，多项指标名列全省前茅。十件民生实事43项任务全面完成，兑现了对市民的承诺。市妇女儿童活动中心动工建设，市老年大学投入使用。圆满完成省国防动员演习任务和征兵工作，大学生兵源占比提升至88.1%。完善军民融合制度，双拥创建不断加强。工青妇幼、档案方志、防震减灾、残疾人、红十字会、打私人防、民族宗教等工作有效推进。

一年来，我们坚持狠抓政府建设，加快构建高效便民、廉洁清正的行政服务体系

着力推动政府工作项目化、实体化。把每项重点工作落实到明确时间、具体项目，确定任务书、施工图，一宗一件推动各项部署落到实处，政府整体运作及效能有了新的提升。2018年政府工作报告共分解工作任务328项，已完成320项，未完成8项，完成率97.6%，比上年提高1个百分点。

着力加强市镇协调联动。推动市民服务中心的改建，整合跨部门办理事项，部门协同进一步加强。推进优化市直管镇体制改革，设立松山湖、水乡新城2个统筹政务服务的功能区。将直接面向人民群众、量大面广的事项依法下放至镇街，基层积极性得到进一步发挥。市镇村三级政务服务体系基本形成，市镇协调联动进入新阶段。

着力提升服务群众水平。谋划推进30个上档次、上规模、高水平的重大民生项目，年内开工17个。启动一体化政务服务平台建设，编辑全市统一的办事指南，公布1462个“最多跑一次”事项清单，简化证明材料200项。与广州初步实现工商、发改等11项业务跨城通办。东莞12345热线在全国334个城市服务评比中排名第9。

着力提升依法行政水平。深入开展宪法主题宣传活动。出台生态文明建设促进与保障条例、饮用水源水质保护条例。严格落实中央八项规定及其实施细则精神，大力整治各种隐性变异“四风”问题。认真办理好人大议案、代表建议和政协提案，加强行政监察和审计监督。基层公务员精气神有效提振，政府职能和作风进一步转变。

各位代表，过去一年，全市上下齐心协力、开拓进取，发展迈出了坚实步伐，成绩来之不易。这离不开上级和市委的正确领导，离不开历届党委政府打下的坚实基础，是全市人民共同奋斗的结果。在此，我代表东莞市人民政府，向全市干部群众，向人大代表和政协委员，向各民主党派、各人民团体、社会各界人士，向各驻莞单位、驻莞部队和武警官兵，向所有参与和支持东莞建设发展的港澳台同胞、海外侨胞和国际友人，表示衷心的感谢和崇高的敬意！

与此同时，我们也清醒地看到，全市发展仍然存在不少困难和问题，需要引起高度重视：一是经济运行的一些短板还比较明显。受发展中不确定因素的影响，经济下行压力加大，生产总值、固定资产投资等指标的完成情况与年度计划目标存在差距，全市科技创新能力、抗御风险能力还不够强。二是污染治理和生态环保任重道远。水污染治理任务依然艰巨，黑臭水体整治任务繁重，大气、土壤、固废等污染防治水平仍需加快提升。三是城市空间需要大力拓展优化。城市发展分散化、碎片化较为严重，开发强度已经逼近极限，“两违”治理欠账较为严重，城市更新的浓厚氛围尚未形成，交通拥堵问题比较突出。四是服务企业水平还不够高。不少企业面临较大的生产经营压力，对企服务政策精准化、个性化水平有待提升，办事手续环节多、时间长的问题仍然存在，企业获得感不够强烈。五是责任意识、进取意识和效率意识仍需进一步强化。政府系统一些干部主动担当、敢于创新、攻坚克难的意识不够强，懒散安逸和不作为、慢作为的现象依然存在，执行效率和工作作风有待强化。对此，我们必须高度警醒，尽最大努力予以有效解决。

2019年工作安排

今年是新中国成立70周年，也是全面建成小康社会的关键之年。机遇与挑战同在、优势与风险并存。机遇体现在我们有以习近平同志为核心的党中央坚强领导，有总书记对广东的一系列重要批示指示精神，为全市工作提供了正确指引和坚强保证。近期中央又不断释放出减税、降费、降准等积极信号，未来发展的环境总体宽松稳定。机遇体现在中央和省正大力推进粤港澳大湾区、广深港澳科技创新走廊等的建设，有效破除行政界限的束缚藩篱，促进资源要素的自由流动。东莞凭借地理区位、产业配套、科技成果转化等方面的独特优势，必将有更大的担当、更大的作为，成为大湾区东西两岸融合发展的主轴。机遇体现在东莞一直是社会各界普遍看好的发展热土，人口保持净流入、市场主体不断增加，城市的活力吸引力不断增强。更有历届党委政府打下的坚实基础、近年来转型升级的扎实成效，全市发展正当其时、未来可期。从外部经济形势来看，我们面临着短期不确定性与中长期挑战叠加的更大考验。当前经济运行稳中有变、变中有忧，外部环境复杂严峻，经济面临下行压力。既存在贸易摩擦持续、企业成本上升等因素影响，也有全市人才支撑不足、镇村组发展动力不够等内生性问题。从区域发展格局来看，我们面临着对标先进城市与建设新一线城市的更高标准。随着雄安新区、海南自贸区等建设的大力推进，区域间的竞争、城市间的竞争越来越激烈。粤港澳大湾区各个城市之间的竞争比拼也在不断加强。对标一流、对标一线，我们不进则是退，小进也是退。从自身发展阶段来看，我们面临着土地空间与财政投入的更严约束。全市可用土地资源和环境承载能力已经逼近极限，过去我们比较熟悉的土地开发、财政投入模式都发生了深刻的变化。只有在绿色发展、集约用地、拓展空间、增收节支等方面多想办法，多出实招，才能打破制约发展的“天花板”。从工作执行落实来看，我们面临着改革再出发与攻坚再加力的更新要求。东莞改革开放已经步入深水区，触及的都是深层利益和矛盾。面对污染治理、“两违”整治等硬任务，面对节能减排、安全生产等硬要求，面对项目落地、“三旧”改造等硬指标，全市各级各部门必须坚持守土有责、守土尽责，拿出舍我其谁的使命感和改革开放再出发的新担当，一个一个项目去落实，一个一个难点去突破，一件一件工作去完成，踏石留痕，久久为功，全面展现新时代新东莞的新作为。

综合研判，未来三年是东莞大有可为的机遇期、黄金期，更是我们推动高质量发展的攻坚期、窗口期。经过三年左右的努力，东莞将迈入万亿GDP俱乐部，市一般公共预算收入逼近800亿元，全市服务企业、保障民生的能力进一步加强，粤港澳大湾区先进制造业中心的名片进一步擦亮，在大湾区和全国发展大局中的地位进一步提升，东莞这座千年古邑、制造名城将焕发出新的夺目光彩。

2019年全市经济社会发展的主要预期目标为：生产总值增长7%，实际执行时努力争取更好结果；人均生产总值增长6.5%；城乡居民人均可支配收入增长7.5%以上；市一般公共预算收入增长6%；国家高企数量达6500家，R&D投入占比提高至2.65%左右；新建截污管网1000公里；空气质量达标天数310天以上；PM2.5浓度控制在35微克/立方米以内。

上周，市委十四届八次全会胜利召开，会议对当前及今后一个时期全市各项工作进行了深入的动员部署，提出了全力打造“湾区都市、品质东莞”的战略任务和价值追求，要求大力实施城市品质提升、发展空间拓展、产业体系升级、基层基础强化、民生福祉增进、重点改革突破等“六大工程”，奋力开创新时代东莞工作新局面。根据这一总体部署，全市政府系统将坚持以习近平新时代中国特色社会主义思想为指导，全面贯彻党的十九大和十九届二中、三中全会以及中央经济工作会议精神，全面贯彻落实习近平总书记对广东重要讲话和对广东工作一系列重要指示精神，深入落实省委十二届六次全会精神和“1+1+9”工作部署，在市委的坚强领导下，以粤港澳大湾区建设为“纲”，突出强化执行、狠抓落实，坚持只争朝夕、马上就办，坚决打赢“九大攻坚战”，着力提升“九大品质”，全力打造“湾区都市、品质东莞”。

一、坚决打赢湾区建设攻坚战，着力提升开放合作品质

把参与粤港澳大湾区建设作为东莞新时代深化改革开放的“纲”，作为推动高质量发展的大机遇、大文章，更加主动地从整个湾区层面来谋划和推动自身发展，着力打造大湾区先进制造业中心、国家科技成果转移转化示范区、全面开放合作先行区、高品质低成本的现代生态都市。

加快融入大湾区一体化交通体系。立足东莞作为大湾区地理几何中心的特点，着力强化交通枢纽功能，积极打造粤港澳大湾区重要节点城市。加快深茂铁路、莲花山过江通道等跨珠江口通道建设，虎门二桥5月1日前建成通车。加快广深第二高铁及始发主站的规划和赣深客专东莞段、佛莞城际的建设，开通穗莞深城轨，抓紧完善“五纵四横六连”的高速路网，构建对接湾区主要城市“1小时交通圈”。大力推进与广州、深圳7条城市轨道的连通，尽快打通与周边城市的断头路，形成与两大中心城市交通互联、产业互动的合作发展新格局。全面开工建设地铁1号线一期，争取开工建设2号线三期、完成3号线一期工程前期工作，加快“8”字形环城路＋多条放射状市域快速路网的建设，构建外引内联的一体化交通网络。

加快打造对接湾区三大战略平台。力争把滨海湾新区打造成为新时期代表东莞参与大湾区建设和对外开放的战略平台。坚持高起点规划、高水平建设，加快集聚高端制造业总部，大力发展现代服务业，加强与港澳台、前海、南沙合作，争取创建成为自贸区新片区，争创更多的金融、税收、产业、人才等政策红利，逐步建设成为国家级重大发展平台。力争把中子科学城打造成为东莞参与国际科技创新中心建设的重要支撑平台。推进散裂中子源二期建设，加快谋划南方光源等大科学装置，对标国家实验室加快建设松山湖材料实验室，打造世界级重大科技基础设施集群。加强与深圳光明科学城、港深落马洲河套地区的协同合作，推进“中子源-光明科学城”主通道规划建设，增强重大科学设施集聚协同效应，携手打造粤港澳大湾区综合性国家科学中心。力争把水乡新城片区打造成为解决发展不平衡不充分问题的新增长极。切实提升水乡管委会在规划制定、土地整备、招商引资等方面的统筹力度，以中心启动区1-2平方公里作为近期重点发展区域，示范带动片区各镇加快发展步伐，使全市经济社会发展的版图更加均衡。

加快建设“城市会客厅”。坚持高起点谋划，按照国际一流湾区和世界级城市群的标准，着力优化城市形态、提高城市档次，加快建设“湾区都市”。坚持高水平规划，着力构建“三心六片”的城市空间格局。坚持高质量建设，着力推动一批市镇重点项目成为精品建筑和品质工程。坚持高品位管理，精心打造好路灯、井盖、路牙石、道路护栏等城市小品、城市家具。聚焦中心城区“一心两轴三片区”建设，优化行政文化中心区的服务能级和综合环境，提升鸿福路、东莞大道的功能品质，实质性启动南城国际商务区建设，加快黄旗南生态科创区、“三江六岸”历史文化区的规划建设，充分彰显“半城山色半城水、一脉三江莞邑香”的山水莞邑特色。聚焦“三大沿线”综合整治，完成广深高速沿线建筑外立面改造和关键节点景观改造，启动广深港高铁、广深铁路沿线“脏乱差”问题整治，更好地展示东莞城市形象。

二、坚决打赢产业升级攻坚战，着力提升经济发展品质

把握经济高质量发展的内在要求，大力推动以先进制造业为核心的实体经济发展，加快培育新的发展动能，推动东莞的经济规模优势尽快转化为集群优势和质量优势。

积极构建面向未来的现代化产业体系。瞄准世界科技前沿，重点布局新一代信息技术、高端装备制造、新材料、新能源、生物科技等新兴产业。顺应5G商业化应用趋势，加快“芯屏机核”等关键技术突破，提升智能手机等优势产业发展水平。力争全市培育出若干个千亿级的新兴产业集群，各镇街形成一批百亿级产业集群。大力发展“院士经济”，引进更多优质科研资源和高新技术项目。结合城市更新，进一步优化商业布局，支持大型零售龙头企业在莞设立法人公司或结算中心，加快发展批发零售业、连锁商业，做强做旺商圈消费。用好虎门港综合保税区、跨境电子商务综合试验区政策红利，大力发展保税进口、保税物流、转口贸易等新模式。“一企一策”支持地方法人金融机构加快发展。积极筹建信用保险公司。紧抓证券发行注册制试点政策机遇，打造更多的上市企业。

积极推动重特大项目招引和落地建设。进一步理顺市镇招商体制，建立一体化专职招商队伍，梳理细化招商目录，出台招商引资实施细则，瞄准跨国公司、大型央企、大型民企和战略性新兴企业等目标，着力引进一批几十亿、上百亿元的重大产业项目。市领导挂点服务5亿元以上的洽谈项目，加快推动项目落地。力争每个镇街（园区）引进2至3个大型产业项目。全年引进超亿元内资项目130宗，超千万美元外资项目100宗。开展重大项目建设“解难题、促开工”攻坚活动，每季度集中开工建设一批重大项目，实行市领导包开工和“小围合”会商机制。挑选一批财政投资项目实行代建制。强化项目履约监管，切实提高履约率、开工率、资金到位率。大力推动新能源电池、正中创新综合体、欧菲影像等90个新项目开工。完成重大项目投资700亿元，力争750亿元。

积极推进国家创新型城市建设。牢固树立“创新是第一动力”的理念，深度参与国际科技创新中心建设，打造广深港澳科技创新走廊的重要支点。推动松山湖向先进高新区看齐，强化创新人才聚集、关键技术研发、战略性新兴产业培育等方面的核心引领作用，更好地建设国家自主创新示范区。推动新型研发机构提质增效，切实提高成果转化和产业培育水平。着力开展“卡脖子”和颠覆性技术攻关，扶持一批研发项目进入国家和省布局。实施百强创新型企业培育计划，争取3年打造100家行业“隐形冠军”。大力引进具有自主知识产权、掌握关键核心技术、项目产业化前景广阔的科技人才（团队）来莞创新创业，积极汇聚全球科技创新成果在莞孵化和产业化。

积极推动制造业转型升级。加快“倍增计划”扩容提质，进一步完善政策体系，力促一批试点企业实现3年倍增。加快建设智能制造全生态链，推进智能加工装备国检中心建设，培育250个以上自动化智能化改造应用项目，认定20个市首台（套）重点技术装备产品，推广使用莞产先进装备。建立工业投资和技改投资工作台账，实行分片包干、分级督导，力争工业投资增长9%。深入推进“筑云惠企”工程，加快工业互联网应用示范，推动100家工业企业上云、10家工业企业实现核心业务数字化。深入推进珠三角大数据综试区建设。力争先进制造业、高技术制造业增加值占比分别达到53%和39.5%。

三、坚决打赢空间拓展攻坚战，着力提升集约发展品质

牢固树立城市空间集约开发理念，力争在土地整备、城市更新、“两违”治理等难点痛点问题上取得突破，全方位优化拓展城市生产、生活和生态空间。

抓紧形成自然资源管理新格局。深化管理体制改革，理顺自然资源保护、空间统一规划机制。摸清山水林田湖草等“家底”，做好第三次全国国土调查。建立健全全市建设密度分区制度，构建疏密有致、适度宜居的城市空间格局。深入推进园区统筹组团发展战略，促进产城融合、连片开发改造，打造产业新城升级版。完善地下空间使用权设定和流转政策，开展空中连廊建设研究，提高空间利用效率和开发强度。严查破坏林业、违法用海等行为。

抓紧实施土地收储整备专项行动。研究出台政策有效破解土地权属和利益问题，推动统筹式、联动式开发。发挥政府逆周期调节作用，制定规划、收储、开发联动的三年滚动计划，完善全市土地收储整备工作机制。探索实行领导挂帅督导制度和分片包干制度，加强对镇街（园区）土地收储的督促考评。市级收储土地1000亩以上，各镇街（园区）力争收储一片500亩以上、整备两片500-1000亩的土地，其中，临深片每个镇整备1-2片1000亩左右的土地。大力推进闲置土地清查整改，盘活存量土地8000亩。大力抓好在库储备土地使用规划，科学制定土地供应出让计划。研究以信托方式整合多方权益，提升土地收储整备效率。

抓紧实施城市更新专项行动。完成市镇专项规划编制。出台新型产业用地政策实施细则，率先在广深高速沿线、临深片区启动一批工改M0项目。出台TOD范围内城市更新项目利益共享机制，推进东莞火车站、虎门高铁站等TOD、TID开发，打造一批标志性更新片区和项目。支持企业或业主提高工业用地容积率，补齐老城区、城中村、城乡结合部等设施短板，进一步完善公共配套。加强相关政策宣讲，吸引更多社会力量与资源参与，争取各类险资以股权、债权等形式支持基础设施建设。大力推进30个重点更新单元建设，力争拉动固投400亿元以上。

抓紧实施“两违”整治专项行动。紧盯治理违建2700万平方米的目标，出台违法建设治理方案，强化属地网格化巡查、交叉检查等机制，强力拆除一批顶风作案、影响恶劣的违建。全面摸清历史遗留违法建筑底数，出台违建分类处理办法，以科学处置公共配套类、生产经营性历史违建为突破口，逐步消化存量违建，确保实现“零增量”、“减存量”。

四、坚决打赢对企服务攻坚战，着力提升营商环境品质

坚持把企业家作为城市最宝贵、最稀缺的资源，全面落实中央和省有关减税降费、扶持小微企业等系列部署，全力以赴为广大企业营造最优质的环境、提供最舒心的服务，切实当好莞商的“贴心娘家”、当好企业的“坚强后盾”。

切实在实施“非公经济50条”方面取得明显成效。加快出台配套细则，构建“1+N”政策体系，加大宣传和辅导力度，打通政策落实“最后一公里”，切实增强企业获得感。完善扶持非公经济工作联席会议制度，将“倍增计划”服务平台扩展为全市企业公共服务平台，覆盖企业从原来的280多家提升到超过1万家。推行重点企业首席服务官，市镇领导挂点服务企业，定期面对面协商问题，提升服务企业的精准度和实效性。组织“百会万企”评议营商环境，全面落实减税降费等惠企政策，努力把东莞打造为服务效率最高、营商环境最优的城市之一。

切实在扶持企业开拓市场方面取得明显成效。建立国际贸易动态预警与应对机制，大力推动“莞货全球行”，支持企业在海外建立品牌展销中心，加快打造华南工业展览之都。常态化开展促消费行动，整合实体商超、住宿餐饮、电商平台等资源，扩大莞货采购和推售力度。全面参与“一带一路”建设，加快建设广东（石龙）铁路国际物流基地，拓展东莞港国际航线，做强外贸邮路通道。抓好两岸冷链物流产业合作试点城市和供应链创新与应用试点城市建设。

切实在解决企业历史遗留问题方面取得明显成效。开展土地历史遗留问题专项清理行动，按照“尊重历史、实事求是、分类处理”原则，健全跨部门协调机制，一宗一件予以妥善解决。开展产权补办专项行动，修订历史遗留违法建筑补办不动产权手续实施方案，推动符合条件的建筑申办不动产登记。开展工厂周边环境专项治理行动，完善公共配套设施，加强治安管理，优化生产生活环境。

切实在营造尊崇优质企业、优秀企业家氛围方面取得明显成效。积极构建“亲”“清”新型政商关系，深入挖掘和弘扬企业家精神，开展优秀企业家表彰活动，展播优秀企业家事迹。实施面向新生代企业家的“接力工程”。建立企业首次轻微违法违规行为容错机制，保护企业和企业家合法权益。深入实施“十百千万百万”人才工程，落实企业高管和高层次人才经济贡献奖励、住房补贴等政策，优化“倍增卡”功能，让企业家和高管人才享受更多优惠待遇。

五、坚决打赢环境治理攻坚战，着力提升生态文明品质

将生态环境保护作为永续发展的根本大计，坚持问题导向、民生取向，以义无反顾、壮士断腕的决心，科学治污、系统治污，全力以赴打赢污染防治这一场硬仗。

努力推动东莞的水更清。市领导挂点督办28条重点河涌治理，918名河长和33名湖长负责全市所有河湖的水环境治理和保护。大力推进重污染河涌整治，完成20条黑臭水体整治。大力推进茅洲河、石马河治理，加大现场指挥部的统筹协调力度，全面完成茅洲河治水工程设施建设并发挥效益，石马河口断面、旗岭断面污染物指标再削减70%。大力提升截污治水能力，开展“打通最后一米”工程，新建截污管网1000公里、验收移交通水1000公里、微支管网1500公里。大力开展排污口整治，推进雨污分流工程。强化海岸线整治修复。完善饮用水源保护区区划。

努力推动东莞的天更蓝。加快推进蓝天保卫战32项强化措施。推进自备电厂煤改气，压减煤炭消费量100万吨以上。基本完成“散乱污”企业整治任务。淘汰国Ⅲ及以下柴油车，实现公交车100%纯电动化。完成燃气电厂及垃圾焚烧发电厂提标改造和生物质锅炉淘汰整治。深入推进VOCs综合整治。强化扬尘污染控制，实施在建工地和拆除工程扬尘管控措施，推动渣土运输车、垃圾收运车等运输全密闭化、规范化。单位GDP二氧化碳排放强度下降5.09%。

努力推动东莞的城更美。推广彩色林建设，打造多层次、多树种、多色彩的城市绿化景观。推进银瓶山森林公园三期、东莞植物园工程（二期）等建设，完成华阳湖国家湿地公园试点验收，争取创建省森林小镇3个，抚育森林1.5万亩。加强固体废物处理处置，编制工业固体废物污染防治规划，启动污水处理厂污泥处置设施扩建工程，建成市区有机资源再生利用一期工程、麻涌餐厨垃圾处理厂（二期），推动立沙岛危险废物综合处置项目试运营。开展重点工业园区土地污染状况调查，建立重点监管企业名录制度。落实耕地“三位一体”综合保护，提升现代农业、观光农业发展水平，保障粮食生产安全。启动国家生态园林城市创建。全力争创国家生态文明建设示范市。

六、坚决打赢城市管理攻坚战，着力提升城市内涵品质

把高品质作为孜孜不倦的追求，对标国内外一流城市，以“匠心精神”和“绣花功夫”加强城市管理，努力提升城市的品位和内涵，持续增强城市的人性化和舒适度。

进一步提升城市精细化管理水平。实施“行走东莞”计划，由市镇领导带队，以巡查抽查的方式，建立不分时段、不打招呼、直奔一线的领导督导机制，开展“地毯式”巡查督导，做到问题当场发现反馈、即时整改落实。实施“厕所革命”专项行动，新建、改造升级1000座公厕，落实“所长制”，全面提升加油站、商场、农村、餐馆等场所厕所保洁水平。实施“洁净城市”专项行动，加强对占道经营、生活噪音等的综合执法，解决背街小巷的环卫保洁常态化问题，推进生活垃圾强制分类，力争6月底前完成20个镇级填埋场整改任务。实施“点睛亮景”专项行动，抓紧推进中心广场、东莞大道、环莞快速路等区域绿化景观提升、城市灯光工程，打造东莞“十里迎宾景观带”。实施“一镇一公园、一村一景点”专项行动，完成第一批50个项目和21个街头小景。实行“大城管”精细化管理，将国省道、农贸市场和砂场管理等纳入考核，实现“数字城管”与公安视频等的全面对接。编制海绵城市规划建设管理办法，完成20个易涝点整治。

进一步提升城市拥堵治理水平。深入开展品质公交创建、学生接送绿色通道、交通秩序整治、停车管理优化、智慧交通建设、魅力慢行提升等11个专项行动。研究缓解东莞大道鸿福路口、几大学校周边、松山湖大道中医院路段、虎岗高速和龙大高速交织路段等重点路段拥堵问题。春节前完成第二批34个拥堵节点治理，梳理开展第三批拥堵节点治理。力争拥堵问题年内有较明显改善。

进一步提升公共安全水平。深化扫黑除恶专项斗争，严厉打击入室盗窃、车内财物盗窃、扒窃诈骗等违法犯罪行为，坚决扫除“黄赌毒”。完善“二标四实”数据采集更新长效机制，加快打造“科技护城墙”，实现精准打防管控，进一步提升群众安全感。加强“智网工程”队伍和机制建设，逐步完善推广至社会治理全领域。严格落实安全生产“四个全覆盖”，加强风险分级管控和隐患排查治理，抓好危化品等行业监管，积极创建安全发展示范城市。开展“打通生命通道”专项整治行动，深入排查治理“三小场所”、出租屋等消防安全隐患，确保村（社区）兼职消防队人员和装备配备100%达标。优化应急管理体制，建设应急综合平台体系，实现对全灾种的全流程、全方位管理。深入推进信访制度改革，依法及时就地解决群众合理诉求。积极创建国家食品安全示范城市。

七、坚决打赢乡村振兴攻坚战，着力提升镇村发展品质

围绕农村经济持续发展、农业高质量发展、打造生态宜居的美丽乡村、乡风文明建设、创新乡村治理、打好脱贫攻坚战等方面，突出具体项目，注重重点突破，努力形成与城市相得益彰的城乡融合发展格局。

着力推动镇村协调平衡发展。完善镇村项目引进利益共享机制，实行资金池竞争性分配，落实好用地倾斜、交通路网优化、债务化解等支持政策，力争8个次发达镇GDP增速快于全市。实施次发达村（社区）创收能力巩固提升行动，力争70个次发达村（社区）经营性纯收入增速快于全市。

着力推动镇村工业厂房改造提升。全面摸清镇村工业厂房底数，建立综合信息数据库。研究出台镇村工业厂房分类改造、土地置换、资金扶持、收益分配等政策，支持镇村集体回购厂房。设立改造专项资金，鼓励村组集体通过自行改造、单一主体挂牌招商等形式，开展旧厂房连片改造。实施工业厂房租赁市场专项整治行动，治理“二手房东”问题，严厉打击囤积厂房、哄抬租金等违规行为。力争每个镇街整合盘活厂房资源20万平方米以上，为科技型创业企业提供更多的低成本空间。

着力推动魅力小城和美丽乡村建设。强化魅力小城建设统筹指导，加快推进万江龙湾等10个示范片区建设，完成南城水濂景观路等28条示范道路提升。出台市域乡村建设规划。全方位推进农村人居环境整治，完善道路照明、停车线、绿化等配套，清理卫生死角，八成村（社区）达到干净整洁村标准。抓好美丽幸福村居建设，力争完成第一批3个特色连片示范项目。

八、坚决打赢公共服务攻坚战，着力提升民生保障品质

把广大群众视为父老乡亲兄弟姐妹，坚持把老百姓拥护不拥护、赞成不赞成、高兴不高兴作为政府工作的出发点和落脚点，做到顺应民心、尊重民意、关注民情、致力民生，让东莞这座城市更有温度、老百姓的幸福更有质感。

深切关注民生民情。对贫困人口、失独家庭、特殊儿童等弱势群体的生活状况给予更多关注和帮助。抓好救助管理站二期建设，调整最低生活保障标准，开展困难家庭重特大疾病“二次救助”，建立失能人员长期照护保险制度，健全特困供养人员护理机制，探索加强对困难群众的兜底性社工服务。实施“一降两升三配套”社保工程，完善全方位医保体系。探索建立搜集、处理、反馈民生诉求机制，市镇领导定期听取、解决群众利益诉求，对民生热点难点问题，第一时间摸底调研，第一时间拿出解决措施，打造“民生大莞家”品牌。

大力补齐“五个位”民生短板。统筹推进优质学校供给，新建扩建公办中小学（幼儿园）85所，完成13所。新提供随迁子女义务教育公办和补贴学位16万个。在农贸市场、医院、学校、公园等停车难区域规划建设一批立体车库，新增一批停车位，缓解“停车难”。加快区域中心医院建设，提升医疗服务水平。新增病床位1640张以上，市人民医院肿瘤中心和市儿童医院新住院大楼投入使用。新建改建养老院3所，新增养老床位500张以上，推广南城篁村“4050”家庭妇女组建居家养老队做法，推进居家养老“大配餐”试点，为60岁以上户籍老人购买意外伤害险。加快高技能公共实训分基地建设，开展粤菜师傅等培训，城镇新增就业岗位8万个。

大力提升教育卫生水平。加快筹建3所高标准未来学校，组建教育集团15个。探索出台政策，激发校长和一线教师积极性。制定民办教育“1+N”政策。扩充公益普惠性学前教育资源。创新职业教育人才培育模式。扩大东莞职业技术学院办学规模。推进东莞理工学院国际合作创新区建设。抓好市和片区青少年活动中心建设。实施公立医疗机构薪酬制度改革。推进医疗服务价格改革，全面取消耗材加成。加快社卫服务机构标准化建设。积极推动市人民医院和市中医院争创省高水平医院。做好国家卫生城市复审迎检。

大力提升市民对东莞的认同感、荣誉感和归属感。着力营造浓厚文化氛围，实施“基层文化百村示范工程”，完善图书馆、文化馆总分馆体系，组织庆祝新中国成立70周年系列活动，办好志交会、漫博会和文采会，加快建设文化名城。着力丰富文体活动，谋划建设一批高水平的文体项目，办好篮球世界杯（东莞赛区）、市民运动会等赛事，推动公共体育设施100%开放，更好地满足市民群众特别是年轻人的需求。着力完善商旅配套设施，建设一批高档次的酒店、民宿。着力发展全域旅游，充分利用好文物古迹和红色文化资源，抓好水乡道滘特色小镇、谢岗银瓶湖湿地公园、塘厦文旅综合项目等建设，打造一批“望得见山、看得见水、记得住乡愁”的乡村游景点。

大力推进对口支援和帮扶工作。高质量完成第八批援疆工作，推动兵团草湖产业园三期40万锭纺织项目建成投产。做好巴宜区11个援藏项目验收工作。推动昭通3个区县脱贫出列。与牡丹江市加强现代农业、旅游康养等合作。做大做强韶关“2+10”园区平台，力争三年引进投资1000亿元。抓好对韶关、揭阳精准扶贫精准脱贫工作，进一步加强驻村力量，财政到村引导资金增加50%。

大力推动其他社会事业发展。办好十件民生实事。加强退役军人管理机构建设，做好全国双拥模范城中期迎检，打造“军民融合创新示范基地”。防范化解房地产市场风险，建立健全房地产市场平稳健康发展长效机制，加大对住房困难群众的保障力度，完善公租房分配、租赁补贴政策，扩大公积金缴存覆盖面。健全扶持机制，引导社会组织有序参与基层治理。办好科普活动，提高全民科学素养。

九、坚决打赢政府建设攻坚战，着力提升行政效能品质

以打造廉洁高效人民满意的服务型政府为目标，全面优化对东莞企业、东莞企业家、东莞市民的各项服务，全力推动政府各项工作马上就办、尽快上马、办就办好，不断提升全市政府系统的执行力和公信力，推动东莞成为珠三角乃至全国行政效率最高、营商环境最优、企业和群众获得感最强的城市之一。

全面加强政府系统党的建设。坚持用习近平新时代中国特色社会主义思想武装头脑、统领政府各项工作，强化党建主责主业意识。严守政治纪律和政治规矩，牢固树立“四个意识”，坚决做到“两个维护”。狠抓执行力建设，强化目标导向、结果导向，不折不扣地将上级和市委各项决策部署落到实处。

全面推进法治政府建设。依法全面正确履行政府职能，提高政府立法质量和规范性文件管理水平。完善依法决策机制，认真倾听民意，推进行政决策科学化、民主化、法治化。推行行政执法公示，强化部门联合执法，规范行政处罚自由裁量权，建立健全权责统一、权威高效的行政执法体制。

全面提升行政服务效率。按照上级部署深化机构改革，加快完成编制划转、部门“三定”等工作。深化镇街（园区）行政管理体制改革，动态调整梳理规范下放事权。推动审批科室向政务服务大厅集中。推广应用莞家政务便民自助终端，实现政务服务事项网上全过程流转。完善市场监管协同创新平台，全面启动“数字政府”和“政务数据大脑”建设，打造“慧治名城”。

全面筑牢反腐倡廉防线。认真贯彻落实中央八项规定及其实施细则精神，严查各种隐形变异“四风”问题，坚决防止反弹回潮。强化对财政资金、国有资产、公共资源、农村集体资产等的监管，加强专项资金的审计监督。依法接受人大及其常委会监督，自觉接受政协民主监督，主动接受社会和舆论监督。严肃整治侵害群众利益的不正之风和腐败问题，推动政府系统党风廉政建设不断取得新成效。

全面提振干事创业精气神。注重运用现场督导、集中整治、专项行动等方式，完善好定期通报、末位约谈、第三方评价等机制，强力倒逼工作落实。建立镇街问题收集、反馈和解决机制。进一步提升镇村基层干部抓规划、抓产业、抓招商的能力和水平，大力整治不作为、慢作为、乱作为的问题。切实抓好年度任务分解和细化落实，确保全年发展目标圆满完成。

各位代表！风正潮平，自当扬帆破浪；任重道远，更须快马加鞭。让我们高举习近平新时代中国特色社会主义思想伟大旗帜，在中共东莞市委的坚强领导下，与全市人民一道，以闻鸡起舞、日夜兼程、风雨无阻的进取心，以逢山开路、遇水搭桥的精气神，凝心聚力，攻坚克难，真抓实干，共同谱写东莞高质量发展新篇章，以优异的成绩向新中国成立70周年献礼！
